# Impact evaluation and association with EuroQol 5D health-related utility values in Ménière’s disease

**DOI:** 10.1186/s40064-015-1527-0

**Published:** 2015-11-24

**Authors:** Ilmari Pyykkő, Vinaya Manchaiah, Hilla Levo, Erna Kentala

**Affiliations:** Department of Otolaryngology, University of Tampere, Tampere, Finland; Department of Speech and Hearing Sciences, Lamar University, Beaumont, TX USA; Department of Behavioural Sciences and Learning, The Swedish Institute for Disability Research, Linköping University, Linköping, Sweden; Audiology India, Mysore, Karnataka India; Department of Otolaryngology, University of Helsinki, Helsinki, Finland

**Keywords:** Ménière’s disease, Quality of life, EuroQol, EQ-5D, ICF, Activity limitations, Participation restrictions

## Abstract

The study was aimed at evaluating the validity of impact measures among patients with Ménière’s disease (MD) with outcome variables of EuroQol generic health-related quality of life (HRQoL) measures (i.e., EQ-5D) by using Visual Analogue Scale (VAS) and EQ-5D index values. 183 members (out of 200 contacted) of the Finish Ménière Association returned the questionnaires that they had filled out. Various open-ended and structured questionnaires focusing on diagnostic aspects of symptoms and impairment caused by the disease were used. For activity limitation and participation restriction, standardized questionnaires were used. Open-ended questions on impact of the disease were asked, and subsequently classified based on the WHO-ICF classification. The general HRQoL was evaluated with EQ-5D index value and EQ VAS instruments. Correlation and linear regression analyses were used to explore the association between HRQoL and other aspects. Based on the explanatory power of different models the disease specific semeionic model provides the most accurate prediction in EQ-5D index calculations (38 % of the variance explained). In EQ VAS scores, HRQoL is most accurately determined by participation restriction (53 % of the variance explained), but the worst prediction was in ICF-based limitations (8 % of the variance explained). Interestingly, attitude and personal trait explained the reduction of HRQoL somewhat better than ICF-based variables. Activity limitation and participation restrictions are significant components of MD, but are less frequently recognized as significant factors in self-evaluating the effect of MD on the quality of life. The current study results suggest that MD patients seem to have problem identifying factors causing activity limitation and participation restrictions and hence use the semiotic description focusing on complaints.

## Background

Ménière’s disease (MD) is commonly approached as an organ specific disease of the inner ear, and is assessed based on vertigo, tinnitus, and hearing loss; although, the behavioral restrictions are far more extensive (Orji 2014). Considering the diversity of this condition, quantifying the impact of disease-related difficulties on measures of quality of life (QoL) and health status utility represents a continuing challenge to researchers.

QoL is the perceived quality of an individual’s daily life, and it can be measured by using standardized instruments. A good QoL in relation to an individual refers to a person managing daily life activities and social relationships well (Williams 1985). The health-related quality of life (HRQoL) is more specific and is related to physical, mental, emotional, and social functioning; however, a health *status* refers to a holistic concept, which is determined by factors which are more than the presence or absence of any disease. It is often summarized by life expectancy or self-assessed health status, and more broadly includes indications of functioning, physical illness, and mental well being. Although the definitions of these two constructs are similar, QOL and health status are distinct constructs (Smith et al. 1999). For example, when rating QOL, patients give greater emphasis to mental health than to physical functioning. However, this pattern is reversed for appraisals of health status, for which physical functioning is more important than mental health (Smith et al. 1999).

The impact of MD can be evaluated by using complaints rated on the basis of severity (Levo et al. 2010), by different impairment questionnaires (Levo et al. 2013), or by using disease specific measures (Stephens et al. 2010; Kato et al. 2004). Various general measures have been used to assess the effect on HRQoL on MD patients (Levo et al. 2012; Anderson and Harris 2001; Soderman et al. 2002; Yardley et al. 2003), but only a few studies have explored the factors associated and resulting in reduced QoL (Levo et al. 2012; Anderson and Harris 2001; Kinney et al. 1997). The disease-specific instruments tend to be more responsive to psychological states and to symptoms of MD, as compared to general health measures that focus on broader aspects of the conditions (Kato et al. 2004; Levo et al. 2012; Diaz et al. 2007). However, the application of general health-related instruments may miss clinically significant changes in QoL in a specific illness because the questions are too broad (Green et al. 2007). Moreover, the QoL measures also seem to be influenced by attitude toward the illness, for example, positive thinking (Stephens et al. 2010). Hence, a more focused approach may be necessary to understand the impact of the disorder.

The World Health Organisation (WHO) has recommended the International Classification of Functioning, Disability and Health (ICF) to be used to describe the complex association among factors such as impairment, functioning, activity limitations, and participation restrictions caused by a disorder on human well-being [World Health Organization (WHO) 2001]. To perform such analysis in MD, Levo et al. (2010) used data from open-ended questionnaires and classified the impairments with the ICF framework. The prediction of impact on QoL was less efficient when using ICF based classification when compared to using impairment questionnaires, which delivered somewhat different explanatory variables (Levo et al. 2013; Stephens and Pyykko 2011). Also, it is important to note that using the ICF framework may provide much broader understanding of the condition’s impact when compared to using disease-specific instruments.

The EQ-5D is a widely used survey instrument for measuring economic preferences for health states. It is one of several such instruments that can be used to determine the quality-adjusted life years associated with a health state. When reporting the general health EQ-5D-3L (3L—referring to three levels in the response scale) results, usually either EQ-5D index value or Visual Analogue Scale (EQ VAS) value has been reported. The index value and VAS evaluations may differ between subjects due to various reasons as dynamic variations of the disease (Bagust and Beale 2005). Other reasons may be due to changes in social communication, personal needs, and acceptance of the impairment. A better knowledge of differences between VAS and EQ-5D index values could help in rehabilitation by providing understanding for the need of proper enablement procedures to restore the quality of life. Moreover, it is also important to understand the relationship between different evaluation approaches (e.g., broad vs focused) on the HRQoL.

The aim of the current study was to evaluate the validity of impact measures among patients with MD with outcome variables of EuroQol generic QoL (i.e., EQ-5D-3L) measures by using VAS and index value instruments.

## Method

### Study design and participants

Permission was obtained from the Finnish Ménière Federation (FMF) to contact their members, asking them to complete an extensive questionnaire on symptoms related to MD. Under Finnish law, this kind of questionnaire study performed in collaboration with patient association does not need ethical approval. For this purpose, every sixth name on their membership list was taken; thus, a sample composed of 200 individuals was contacted. They were sent a 26-page questionnaire by mail as in our previous studies (Stephens et al. 2010, 2012), together with a stamped and addressed envelope for their responses. Those not responding within 12 weeks were sent reminders. Every returned questionnaire was examined; if there were missing data, the respondent was contacted by telephone and asked to answer the unanswered questions so as to achieve complete data. In total, 186 out of 200 sent questionnaires were returned, resulting in a return rate of 93 %; however, 3 questionnaires had significant amount of missing values and were removed. The 183 participants had the mean age of 61.5, and there were 36 men and 147 women in the sample, reflecting the gender spread in FMF.

### Questionnaires

The total questionnaire comprised the Vertigo Questionnaire (Kentala 1996), the EQ-5D-3L measure (Rabin and de Charro 2001), the International Tinnitus Inventory (ITI; Chéry-croze and Collet 2005), The Hearing Disability and Handicap Scale (HDHS; Philibert 1994), Localization questions based on the Hearing Measurement Scale (HMS; Chung and Stephens 1983), a Dizziness Handicap Questionnaire (DHQ; Yardley et al. 1992), a Participation Restriction Scale (Stephens 2001), and the Sense of Coherence (SOC) Scale-Short version (Antonovsky and Sagy 1986). There were also some open-ended questions.

The specific restriction and function limitation open-ended question was worded as follows: *Please make a list of the main effects that your Ménière’s disease has on your life.Write down as many as you can think of.* In this task, five lines were indicated for each subject to fill. Thereafter, the items were classified based on ICF classification [World Health Organization (WHO) 2001]. The classification was done independently by two researchers. However, four researchers discussed the analysis and a consensus was achieved in relation to ICF codes. From the 183 subjects, 176 reported some effects of MD that resulted in some functional limitation. The classification provided 64 different entities belonging to 6 main categories (Levo et al. 2010).

The individuals were asked to rate the impact of MD by asking, “*How much does Ménière’s disease influence in your life?”.* The question was scaled in five steps ranging from *not at all* to *very severely*. This question was used as an outcome measure of disease specific impact of MD on life. In addition, the effect of cardinal symptoms on MD, as vertigo, gait, hearing, tinnitus, pressure in the ear, hyperacusis, and possible other disorders were also rated in a five step scale from *no effect* to *very severe effect*.

In modelling of impairment related to MD and its restrictions, we used EuroQol general health measure, the EQ-5D. The EQ-5D instrument consists of two parts: five questions relating to the distinct dimensions of a patient’s functional capacity mobility, self-care, usual activities, pain/discomfort, and anxiety/depression) on each of which 3 responses are possible, and a visual analogue scale (VAS) on which the patient is asked to indicate a self-rating of their current health state. The former are combined with weightings derived from a sample of the general European population to provide a *‘social tariff’* EQ-5D index value (Dolan et al. 1995). The latter constitutes a more direct indicator of patients’ own implicit preferences. You can find more information about the questions and rating scale used in the EQ-5D by visiting their website (http://www.euroqol.org/).

### Data analysis

The association between EQ-5D-3L and other aspects (e.g., symptoms, activity limitations, etc.) was analysed first by exploring associations with the Pearson correlation matrix and then by using the linear regression analysis method. In EQ-5D index value, each state of the five health-related dimensions is assigned at the three functional levels of no problem, some problem, and extreme problem.

## Results

For the whole population, the average EQ-5D index value values and EQ VAS values are shown in Table 1. It also provides the percentage of study samples in three functional levels in the five dimensions of the EQ-5D.

**Table 1:** The demographic details and summary of EQ-5D quality of life values

Parameter	Mean (±SD)	Lower range	Upper range
Age (in years)	61.5 (10.5)	22	91
Symptom duration (in years)	18.4 (11.1)	1	63
EQ-5D Index value	0.75 (0.19)	0.29	1.0
EQ-5D VAS value	71.2 (17.1)	20	100

	No problem	Some problem	Extreme problem
*EQ*-*5D Dimensions* (% of population reporting the problem)			
Mobility	59.6	40.4	0
Self-care	97.3	3.7	0
Usual activities	74.3	33.4	3.3
Pain/discomfort	44.8	50.8	4.4
Anxiety/depression (mood)	76.5	22.4	1.1

**Fig. 1:**
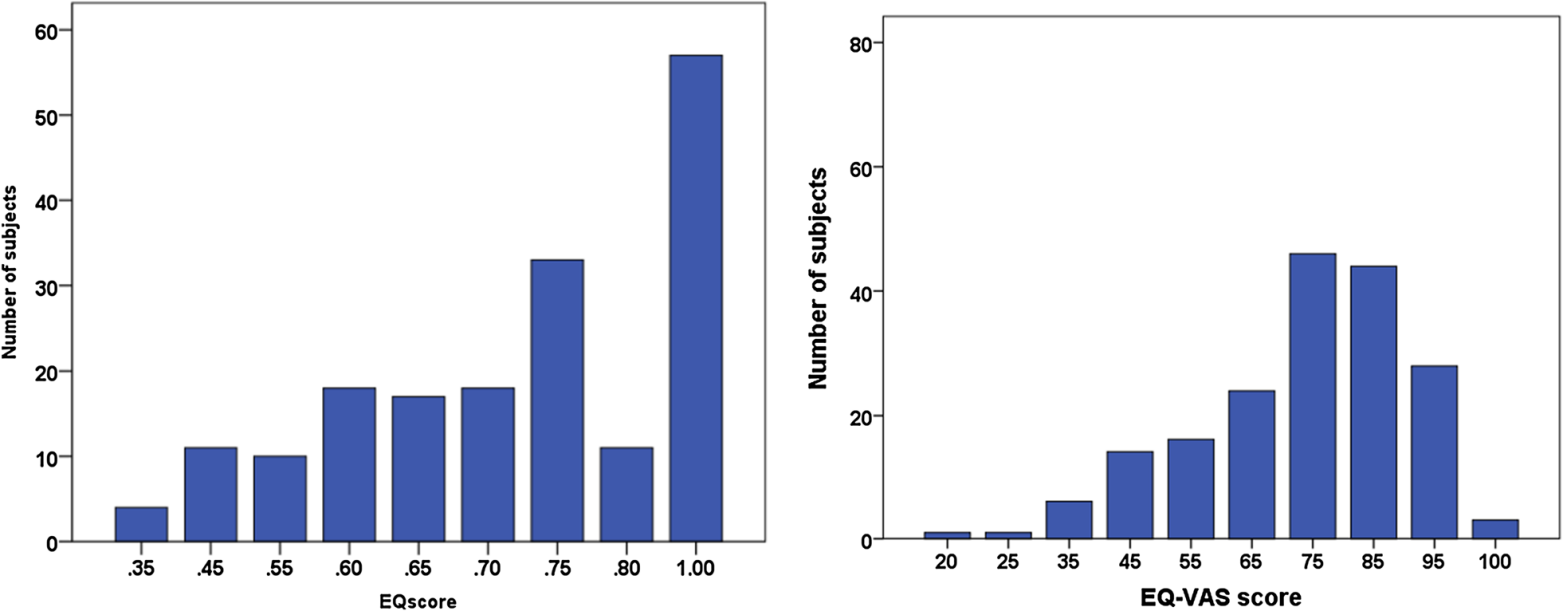
Distribution of EQ-5D Index values (*left*) and VAS scores (*right*) among 183 participants

Figure 1 presents the EQ-5D index value and EQ-5D VAS values in the current sample suggesting skewness in the EQ-5D index value values, whereas EQ VAS values are dominated by an even tenth value in the scale.

**Fig. 2:**
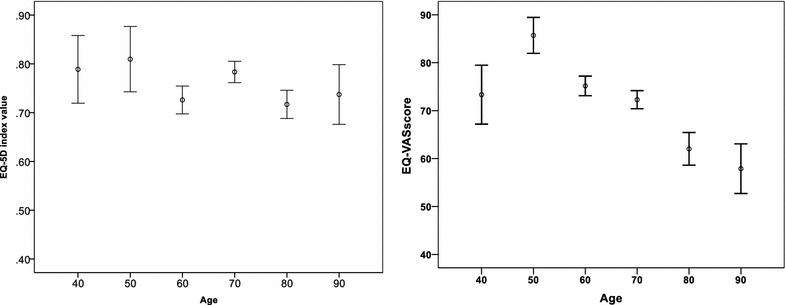
Linear regression model outcome of age in EQ-5D index values (*left*) and VAS scores (*right*)

When individual scores are aggregated for a population sample, the resultant function is inherently nonlinear. Figures 2 demonstrates the EQ-5D index value and VAS values of different age groups. In EQ-5D index values, the effect of age was not significantly dependent on age (F = 1.206, *p* = 0.305). However, in the health score evaluation on VAS scale in the age group of 50 years, the VAS values differed significantly from older age groups (F = 4.401, *p* < 0.01), with older age groups scoring worse VAS values. We therefore standardized the effect of age in linear regression analysis when the VAS instrument was the outcome variable.

### Internal association of EQ score and its association with various symptoms and complaints associated with MD

Table 2 presents correlation results of various symptoms and components of MD with five dimensions of the EQ-5D dimensions. In correlation analysis, motility correlated with usual activities (0.55, *p* < 0.001) and pain (*r* = 0.33, *p* < 0.001). The mood correlated with self-care (0.20, *p* < 0.001), usual activities (*r* = 0.25, *p* < 0.001), and pain (*r* = 0.32, *p* < 0.001). The self-care correlated with usual activities (*r* = 0.29, *p* < 0.001) and mood (*r* = 0.20, *p* < 0.001). Only motility was biased by ageing of the subjects with older subjects having more problems with mobility. Males also had more pain-related complaints than females. The duration of disease did not correlate with any of the QoL components.

**Table 2:** Correlation of EQ-5D components with various aspects of the Ménière’s disease including symptoms of the disease, activity limitations and participation restrictions, personal traits, and attitude

	Motility	Self-care	Usual activities	Pain	Mood
Vertigo severity	*n.s.*	0.19*	*n.s.*	*n.s.*	*n.s.*
Nausea AND vomiting	*n.s.*	0.15*	*n.s.*	*n.s.*	*n.s.*
Tumarkin attack	0.184*	*n.s.*	0.252**	0.216**	*n.s.*
Balance problems	0.496**	*n.s.*	0.451**	0.258**	*n.s.*
Unsteadiness	0.506**	0.198**	0.514**	0.321**	*n.s.*
Moving ability	0.559**	0.357**	0.446**	0.386**	*n.s.*
Chair rise	0.442**	0.271**	0.381**	0.350**	0.176*
Impact of vertigo	0.249**	0.158*	0.272**	0.236**	*n.s.*
Tinnitus impact	0.156*	*n.s.*	*n.s.*	0.125	*n.s.*
Balance impact	0.569**	0.327**	0.433**	0.371**	0.225**
Hearing impact	*n.s.*	*n.s.*	*n.s.*	*n.s.*	*n.s.*
Pressure impact	0.226**		0.229**	0.214**	0.198**
Anxiety/nervousness	0.181*	*n.s.*	0.219**	0.214**	0.349**
Energetics/fatigue	0.286**	0.159*	0.356**	0.344**	0.316**
Sense of coherence	−0.185*	−0.182*	−0.264**	−0.272**	−0.417**

*p ≤ 0.05; **p ≤ 0.01

### Quality of life measured with ICF oriented approach

Table 3 demonstrates linear regression model with the ICF-based limitations when EQ-5D index value and VAS are outcome variables. In VAS evaluation, the model consisting from vertigo, hearing loss, and a salt-free diet was statistically significant (*r* = 0.293, *p* < 0.001) and explained only 8.6 % of variance in VAS. In EQ-5D index value model, two variables were significant: impairment dietary impact and nausea. Also, this model was statistically significant (*r* = 0.257, *p* < 0.001) and explained 6.6 % of variance in EQ-5D index value.

**Table 3:** Linear stepwise regression analysis of ICF-based limitations by the Ménière’s disease with EQ-5D VAS and EQ-5D index values as outcome variables

Variable	VAS model, *r* = 0.293			EQ-5D index value model, *r* = 0.293		
	Coeff	S.E.	*P*	Coeff	S.E.	*P*
Constant	69.1	1.7	<0.001	0.78	0.02	<0.001
Vertigo	−7.6	2.9	0.010	*n.s*	*n.s*	0.168
Hearing	6.9	3.9	0.012	*n.s*	*n.s*	0.192
Diet	0.7	4.2	0.022	*n.s*	*n.s*	0.996
Nausea	*n.s*	*n.s*	0.543	−0.13	0.07	0.011
Drugs	*n.s*	*n.s*	0.161	0.22	0.05	0.003

### Quality of life measured with symptoms oriented approach

Table 4 demonstrates a linear regression model consisting of symptoms when EQ-5D index value and VAS instruments were outcome variables. In VAS evaluation, the model consisted of gait problems, balance problems, physical strain induced vertigo, and shortage of energy. This model was statistically significant (*r* = 0.624, *p* < 0.001) and explained 38.9 % of the variance in VAS. In EQ-5D index value model, three variables were significant: impairment of gait, balance problems and shortage of energy. Also, this model was statistically significant (*r* = 0.634, *p* < 0.001) and explained 37.8 % of the variance in EQ-5D index value. Noteworthy was that neither hearing problems nor vertigo correlated with quality of life in this study.

**Table 4:** Linear stepwise regression analysis of symptoms of the Ménière’s disease with EQ-5D VAS and EQ-5D index values as outcome variables

Variable	VAS model, *r* = 0.624			EQ-5D index value model, *r* = 0.558		
	Coeff	SE	*P*	Coeff	SE	*P*
Constant	84.8	1.8	<0.001	0.90	0.02	<0.001
Balance	−3.8	1.6	0.017	−0.26	0.11	0.019
Gait	−7.6	2.1	<0.001	−0.79	0. 14	<0.001
Physical strain	−3.3	1.3	0.013	*n.s.*	*n.s.*	0.879
Energy	−2.5	1.3	0.048	−0.06	0.01	<0.001

### Quality of life measured with activity limitations oriented approach

For assessment of activity limitations, tinnitus was assessed on a ITI questionnaire with 8 questions. The functional limitation of hearing was assessed by a questionnaire consisting of 10 questions. Localization of sound was assessed by a sound localization questionnaire consisting of 4 questions. The vertigo was evaluated with a vertigo handicap questionnaire consisting of 8 questions. A total of 24 questions were analyzed.

Table 5 presents the linear stepwise regression analysis of factors describing activity limitations in MD on VAS and EQ-5D index based measures of 5D QoL. The VAS instrument based model consisted of two vertigo linked variables (walking on the sidewalk, bending provoked vertigo, and activity limitation by fear of having an attack), one tinnitus related variable (frequency of unbearable tinnitus), and one hearing linked variable (problems of hearing a doorbell). The model was statistically significant (*r* = 0.622, *p* < 0.001) and explained 38.7 % of the variance in VAS. The EQ-5D index value could be explained by vertigo-linked variables (temporary activity limitations and walking in open space), and one tinnitus linked variable (frequency of unbearable tinnitus). The model was statistically significant (*r* = 0.558, *p* < 0.001) and explained 29.6 % of the variance in EQ-5D index value.

**Table 5:** Linear stepwise regression analysis of factors describing activity limitations caused by the Ménière’s disease with EQ-5D VAS and EQ-5D index values as outcome variables

Variable	VAS model, *r* = 0.622			EQ-5D index model, *r* = 0.558		
	Coeff	SE	*P*	Coeff	SE	*P*
Constant	91.8	2.9	<0.001	0.94	0.03	<0.001
Problems with gait on sidewalk	−6.8	1.6	<0.001	−0.05	0.02	<0.032
Unbearable tinnitus occurrence	−3.9	1.1	0.001	−0.04	0.02	0.020
Bending provoking vertigo	−7.4	1.7	<0.001	-0.05	0.02	0.006
Hearing door bell ringing	−3.1	1.3	<0.05	*n.s.*	*n.s.*	0.522
Activity limitation (e.g., shopping) caused by vertigo	*n.s.*	*n.s.*	0.144	−0.071	0.022	0.002

### Quality of life measured with participation restrictions oriented approach

The questionnaire focuses on participation restrictions (consisting of 30 questions) caused by health condition on conversations, traveling, shopping, traveling to medical appointments, banks and offices, attending learning circles, relationship to others, work related activities,among others. Table 6 presents the linear stepwise regression analysis of factors describing participation restriction in MD on VAS and EQ-5D index based measures of 5D QoL. In VAS based model of quality of life, six variables turned out to be indicative (see Table 6). The model was statistically significant (*r* = 0.580, *p* < 0.001) and explained 33.7 % of VAS variability. In EQ-5D index value based model, only three variables were included in the model that was statistically significant (*r* = 0.457). EQ-5D index value, *p* < 0.001) could explain 17.3 % of the variability of EQ-5D index value. In factorial analysis, the participation restriction consisted of 7 factors covering 68 % of the data. Broadly, the variables associated with VAS-measure consisted of communication, listening, daily activities, social activity, and learning restrictions. The EQ-5D index value associated variables consisted of restrictions in social activity and in daily activity. In VAS measure the hearing impairment associated restrictions dominated, whereas in EQ-5D index value the participation restriction consisted mainly from balance impairment associated problems.

**Table 6:** Linear stepwise regression analysis of factors describing participation restrictions caused by the Ménière’s disease with EQ-5D VAS and EQ-5D index values as outcome variables

Variable	VAS model, *r* = 0.580			EQ-5D index model, *r* = 0.457		
	Coeff	S.E.	*P*	Coeff	S.E.	*P*
Constant	109.7	4.04	<0.001	0.89	0.07	<0.001
Participating in lectures	−5.16	1.66	0.007	*n.s.*	*n.s.*	0.968
Restriction on performing household tasks	−5.50	1.89	0.036	−0.114	0.03	<0.001
Hearing quiet conversation	−5.38	1.92	0.006	*n.s.*	*n.s.*	0.444
Problems in visiting doctor	−6.22	2.15	0.005	*n.s.*	*n.s.*	0.917
Loss of interest in watching TV	−5.78	1.91	0.003	*n.s.*	*n.s.*	0.734
Restriction on relationships to close people by hearing problem	8.22	2.23	<0.000	*n.s.*	*n.s.*	0.40
Restriction on visiting close people by hearing problem	−6.74	1.90	0.001	*n.s.*	*n.s.*	0.223
Traveling alone	*n.s.*	*n.s.*	0.547	−0.34	0.02	0.059
Problems staying home alone	*n.s.*	*n.s.*	0.633	0.138	0.62	0.029

### The difference between EQ-5D index value and VAS measurement

Table 7 presents the summary from the feasibility of different parameter descriptions in the evaluation general HRQoL with EQ-5D index value and VAS instruments. For comparison, a model consisting of personal traits measured with SOC and self-rated anxiety is included. Based on the explanatory power of different models, it seems that the disease symptom specific semeionic model provides the most accurate prediction in EQ-5D index value calculations (37.8 %). In VAS scores, QoL is most accurately determined by participation restriction (53.3 %). The worst prediction in both EQ-5D index value and VAS models was in ICF-based limitations (5.6 and 7.9 % respectively). Interestingly enough, attitude and personal traits explained the reduction of QoL somewhat better than the ICF-based variables.

**Table 7:** Goodness of fit models describing health related quality of life with EQ-5D index value and VAS instruments when evaluated with ICF-based limitations, symptom specific complaints, activity limitations, and participation restrictions variables

Measurable	VAS (%)	EQ-5D index value (%)	Ménière impact (%)
ICF-based limitations	8.6	7.9	8.7
Symptoms	38.9	34.2	48.3
Activity limitations	38.7	32.1	47.2
Participation restrictions	33.7	17.3	53.4
Attitude and personal trait	8.8	22.7	23.4
Symptoms, attitude and personal trait	39.2	44.8	51.1

For comparison, attitude and personal trait measures are included

## Discussion

The aim of the present study was to evaluate the validity of impact measures among patients with MD with outcome variables of EuroQol generic QoL. Furthermore, differences between two generic health evaluation methods in EQ-5D (EQ-5D index value and VAS) were explored. We found that the symptom profile of the disease provided the major outcome in generic HRQoL. The VAS instrument had seemingly an inherent property to include age-associated changes in performance as well as in attitude and personal trait. If these variables were added in the VAS-instrument that contained the symptom profile, the regression (38.9–39.2 %) did not significantly improve. In contrast, the EQ-5D index value -instrument was markedly improved by attitude and personal trait (i.e., 37.8–45.3 %). As these measures are not within the EQ-5D index value –instrument, the VAS-instrument provides different aspects of QoL in MD, which can be missed if only one instrument is inspected. The attitudes towards the disease and personal trait played a minor role in the estimation of QoL in MD, which is consistent with results from previous studies by Levo et al. (2012) and Stephens et al. (2010). Vigor and energy are not normally explored in relation to MD, although the patient often complains about a lack of energy (Stephens et al. 2010). This variable could partly explain the reduction in QoL, and the difference in EQ-5D index value and VAS scaling methods. Values and value judgments are intrinsic to measurements of this sort and need to be made explicit. The results confirm the previous observation that there is a shortage of relevant and validated questionnaires assessing the impact of vertigo or dizziness on generic QoL (Duracinsky et al. 2007).

Correlation analysis indicated that mood (e.g., anxiety/depression) was related to very few aspects of complaints and symptoms, whereas the other four dimensions of the EQ-5D (mobility, self-care, usual activities and pain) were associated with more complaints and symptoms of MD (see Table 2). In addition, various internal correlations were observed (e.g., motility correlated with usual activities and pain; mood correlated with self-care, usual activities, and pain; self-care correlated with usual activities and mood). Moreover, some age and gender effects were also noticed, although the duration of the disease does not seem to be related to any of the QoL components. These observations provide useful information for clinicians in management and rehabilitation planning of MD patients. This indicates that learning to cope with the disease (Kentala et al. 2013) does not necessarily improve QoL as has been suggested (Tyrrell et al. 2015).

In the present study, the individuals with MD tend to evaluate their health with a symptoms based approach rather than with the limitation of function. Control of symptoms may be a more understandable way to improve the QoL and influence the instrument values when compared to the effects of limitations and various restrictions experienced by the patient. In this respect, our observations confirm the concept that condition specific symptom measures that mirror treatment or condition of certain illness have high acceptability and relevance for patients and doctors (Kind 2001). They also can be influenced by treatment, and are sensitive to change. In the therapeutic process, interest has therefore been focused to change the medical factors reducing the EQ-5D index value and VAS scores. However, as indicated in the present study, not all the items are related to medical conditions. Some of the VAS and EQ-5D index value scores are linked to personal trait and attitude. Personal trait is, however, resistant to changes as SOC is difficult to change in adult subjects. However, possibly some attitude dependent variables in activity limitation and participation restriction can be influenced by therapy as shortage of energy, ability to drive a car, capability to do shopping, and the uncertainty with management at work. If medical conditions cannot be alleviated, then the rehabilitative efforts might be focused on these domains in order to improve accessibility and remove hindrances. One such effort is a peer support system, involving patient-to-patient help, and also support from significant others can be enhanced.

The WHO-ICF is a multipurpose classification designed to serve various disabilities and health conditions [World Health Organization (WHO) 2001]. It specifically aims to provide a scientific basis of understanding through studying health and health related status, outcomes and determinants. ICF can be used as an explanatory framework that may allow more comprehensive understanding of the character of illness, and how it may be described and potentially alleviated (Wade and Halligan 2003). The ICF provides a patient-centered illness description that may provide useful insights into finding solutions to overcome the impact of the condition. It was, therefore, interesting to study the applicability of ICF to be used in a model describing QoL. With EQ-5D index value and VAS value, the ICF based limitations provides the possibility to examine differences from patients’ perspectives and compare them with the perspectives of the observer. Although the VAS and EQ-5D index value based models yielded rather poor explanatory power (8 %), the limitations loaded partly differently into the EQ-5D index and VAS models. The poor performance in connection with QoL instruments may be due to two factors: (1) patients had problems identifying items limiting their activities when asked open-ended questions; or (2) alternatively they had adapted to their current situations and had thus not identified existing problems if not specified by a questionnaire. The former option might occur due to temporary changes in the disease, as patients tend to focus on more recent symptoms. The latter option would occur if the measures of QoL would reflect other aspects of the illness as, for example, *own will* that is not directly described in ICF-classification. Both of these aspects may be true and should be evaluated in further studies. One way of doing that might be to understand how the ICF-based approach will relate to QoL measures when used with open-ended and structured questionnaires. However, the current study results indicate that the patients were not able to identify the crucial factors describing the illness, or that illness has more dimensions than defined by ICF.

In MD, the VAS values seem to contain additional items that will confound the evaluation of the impact of the disease. Such confounders in the present study were mood, attitude, expectations of progress of the disease, and ageing among others. We also observed that VAS includes some important items as cognitive ability, memory, vitality, and a large scale of social restrictions that were not met in EQ-5D index value -instrument. In MD, the EQ-5D index value measures complaint associated reduction of activity, and is adapted to the ageing process. The EQ-5D index value and VAS are measuring thus somewhat different dimensions of the impact on general HRQoL in MD. The ICF-based items described by the patients did not contain elements that could explain reduction of QoL in the present study. This seems to be due to heterogeneous responses of the patients exhibiting a large scale of different topics in their replies.

## Conclusions

The current study suggests that a more focused symptom oriented approach is more sensitive in relation to general HRQoL, whereas the more comprehensive ICF-based approach explained less variance. The study identified differences between VAS and EQ-5D index value, indicating that they may assist in understanding different aspects of QoL. Overall, these findings suggest that MD patients seem to have problems identifying factors causing activity limitation and participation restrictions and use the semiotic description focusing on complaints.
